# Editorial: Positive Neuroscience: the Neuroscience of Human Flourishing

**DOI:** 10.3389/fnhum.2020.00047

**Published:** 2020-02-28

**Authors:** Feng Kong, Aaron Shain Heller, Carien M. van Reekum, Wataru Sato

**Affiliations:** ^1^School of Psychology, Shaanxi Normal University, Xi'an, China; ^2^Department of Psychology, University of Miami, Coral Gables, FL, United States; ^3^School of Psychology and Clinical Language Sciences, University of Reading, Reading, United Kingdom; ^4^Kokoro Research Center, Kyoto University, Kyoto, Japan

**Keywords:** positive neuroscience, flourishing, well-being, character strengths, neuroimaging

The burgeoning subfield of neuroscience focused on salubrious attributes of the human condition has begun to illuminate the complex biological basis of human functioning and flourishing. This has been referred to as positive neuroscience. Instead of focusing on pathology, research on positive neuroscience directs its attention on the neural mechanisms supporting flourishing, psychological well-being, resilience, and promotion of health. Previous studies have investigated the structural and functional neural basis underlying positive human functioning such as well-being (e.g., Van Reekum et al., 2007; Heller et al., 2013; Kong et al., 2015a; Sato et al., 2015), meditation (e.g., Cahn and Polich, 2006; Sperduti et al., 2012), optimism (e.g., De Pascalis et al., 2013), resilience (e.g., Kong et al., 2015b, 2018), and creativity (e.g., Fink et al., 2009), based on experimental and self-reported measures. However, this emerging literature is just the tip of the iceberg on the quest to identify the complex mechanisms of brain structure and function supporting human behavior. The Research Topic “Positive neuroscience: the neuroscience of human flourishing” provides an outlet for novel work in this domain and to advance our understanding of the underlying mechanisms of aspects of human flourishing.

Kress and Aue begin this topic with a behavioral study on the effect of attention bias modification on optimism bias—that is, being overly optimistic—for future positive events. They found that extensive training in which subjects were required to direct attention to positive social information could enhance comparative optimism bias for future positive events, over, and above trait optimism.

Wang et al. used an activation likelihood estimation (ALE) meta-analysis of functional magnetic resonance imaging (fMRI) studies to investigate whether neural systems involved in prosocial behaviors and reward demonstrated overlapping or distinct neural signatures. They found that prosocial behaviors specifically activated the insula, temporal lobe, and superior temporal gyrus (STG), whereas reward specifically activated the lentiform nucleus, thalamus, caudate nucleus, parahippocampal gyrus (PHG), and anterior cingulate cortex (ACC). Relatedly, Tunison et al. more specifically report on an event-related potential (ERP) component associated with reward processing, the reward-related positivity (RewP). The RewP is a positive deflection ERP component observed between 250 and 350 ms after reward feedback over fronto-central electrode sites, and its amplitude has been related to internalizing psychopathology. However, the RewP has been examined almost exclusively in response to financial rewards, and whether this ERP component is a general feature of reward processing remains uncertain. To address this, Tunison et al. used a point-based system of reward and found that RewP amplitudes were indeed larger for rewarded trials vs. non-rewarded trials. These data add to a growing literature that there are general properties of incentives regardless of the reward type.

Three studies in this issue focused on the neural basis of positive emotion and well-being. First, Hu et al. used functional near-infrared spectroscopy (fNIRS) to examine the brain's hemodynamic responses to different positive emotions. They found 10 typical kinds of positive emotions (joy, gratitude, serenity, interest, hope, pride, amusement, inspiration, awe, and love) could be divided into three distinct clusters (i.e., playfulness, encouragement, and harmony) and hemodynamic responses to these three clusters showed distinct patterns. Second, using fMRI, Hong et al. explored the neural basis of a specific and rarely examined positive emotion type—professional pride. They found that professional pride may be associated with multiple brain networks including the right ventrolateral prefrontal cortex (VLPFC), left dorsolateral prefrontal cortex (DLPFC), left middle and inferior temporal gyri, left posterior superior temporal sulcus, right temporoparietal junction, left lingual gyrus, left calcarine cortex, right insula, left caudate, and right putamen. Third, Goldbeck et al. used resting state fNIRS to investigate neural basis of well-being. Performing a voxelwise regression, they found that the networks linked to individual differences in well-being included areas of the posterior default mode network. Interestingly, they found specific divergence in neural circuits linked to eudaimonic well-being, defined as a sense of meaning and purpose, positive social relationships, mastery, autonomy, virtues, and subjective well-being, a more general term referring to the various types of subjective evaluations of one's life, including both cognitive evaluations, and affective feelings (Diener et al., 2018). Specifically, they found that while the left middle temporal/fusiform gyrus was a hub node of a network associated with eudemonic well-being, the left primary/secondary somatosensory cortex was a hub node of the network associated with subjective well-being. Continued work exploring whether eudaimonic and subjective well-being are linked to distinct neural circuits and relevant health outcomes will be essential to characterizing the specific neural systems associated with eudaimonia versus subjective well-being.

Furthermore, several papers in this issue centered upon the neural basis of positive personal characteristics such as trait mindfulness, creativity, and emotional intelligence. For example, Parkinson et al.  found that trait mindfulness and its facets was associated with increased functional connectivity (FC) in regions linked to attentional control, interoception, and executive function, and decreased FC in regions linked to self-referential processing and mind wandering. In another study, Arkin et al. demonstrated that musical creativity was negatively associated with gray matter volume in the right inferior temporal gyrus and bilateral hippocampus. Motivated by Thayer et al.'s Neurovisceral Integration model (e.g., Thayer and Lane, 2009; Smith et al., 2017) which proposes a key role for brain networks supporting cognitive and affective flexibility in cardiac vagal control, Vanuk et al. assessed the association between emotional intelligence and cardiac vagal control. The authors found that ability emotional intelligence, but not mixed emotional intelligence, was positively associated with cardiac vagal control.

Beyond positive personal characteristics, four studies explored the effect of mindfulness/meditation training on the brain and psychological functions. First, using a two-stage mindfulness training over eight weeks, Zhang et al. examined whether the effects of different components of mindfulness meditation training differentially affected anxiety, depression, and rumination. They found that the first 4-weeks of focused attention (FA) meditation could improve self-reported levels of mindfulness and reduce levels of anxiety and depression, while the subsequent 4-weeks open monitoring (OM) meditation could further improve the level of mindfulness and maintain a positive mood. Second, Kwak et al. explored the neural mechanisms underlying the effect of a 4-days meditation intervention on stress resilience using resting state fMRI FC. They found that increased resting-state FC between the left rostral ACC and the dorsomedial prefrontal cortex (dmPFC) was linked to neural basis of the effect of the meditation intervention on stress resilience that was assessed via the Resilience Quotient Test (RQT). Third, Tang et al. reviewed key components and potential brain-body mechanisms related to well-being and proposed mindfulness training as a promising method to improve well-being. Finally, Reddy and Roy further reviewed the role of one's motivation to engage in meditation practices. They proposed that while practicing meditation one may benefit from traditional assistance and ethical/moral teachings in addition to meditation training in isolation.

Interestingly, several papers in this issue also report on the neural components linked to other positive activities (e.g., expressive writing, martial arts, attention training) that can promote positive human functioning. For example, DiMenichi et al. investigated the effect of expressive writing on neural processing during learning. A large literature finds that expressive writing is linked to healthy psychological function (e.g., Pennebaker and Chung, 2007). Here, they found that writing about a past failure led to increased activation in the mid-cingulate cortex (MCC) during the learning task. In addition, Fujiwara et al. investigated the effect of a form of martial arts, “Kendo,” on the motivation network during attention processing. They found that Kendo players (KPs) exhibited a lower FC between the nucleus accumbens and frontal eye field (FEF) within the motivation network and a higher FC between intraparietal sulcus (IPS) and precentral gyrus (PCG) within the motivation network than non-KPs. Song et al. reviewed the underlying psychological and neural mechanisms that may underlie positive illusions and proposed that increasing positive illusions may be a promising way to improve relationships. Lastly, Zhu et al. used real-time fMRI neurofeedback (rtfMRI-NF) to investigate the capacity to self-regulate hippocampal activity. They found that hippocampal activity and amygdala–hippocampus connectivity can be regulated using rtfMRI-NF.

Finally, Takeuchi et al. investigated the effects of family socioeconomic status (SES) on brain structure. They partly replicated previously observed main effects of family SES on regional gray matter volume and fractional anisotropy. They also observed a significant interaction between sex and family SES in white matter tracts between areas such as the thalamus, corpus callosum, ACC and lateral PFC. The precise pathways by which these effects may manifest are open to debate.

In summary, the articles presented in this Research Topic provide a valuable insight into understanding the biological bases of positive human functioning and flourishing, and highlight new and exciting directions for the field of positive neuroscience.

## Author Contributions

All authors listed have made a substantial, direct and intellectual contribution to the work, and approved it for publication.

### Conflict of Interest

The authors declare that the research was conducted in the absence of any commercial or financial relationships that could be construed as a potential conflict of interest.
